# Outbreak of intestinal amoebiasis among men who have sex with men, Barcelona (Spain), October 2016 and January 2017

**DOI:** 10.2807/1560-7917.ES.2017.22.30.30581

**Published:** 2017-07-27

**Authors:** Laura Escolà-Vergé, Maider Arando, Martí Vall, Roger Rovira, Mateu Espasa, Elena Sulleiro, Pere Armengol, Francesc Zarzuela, María-Jesús Barberá

**Affiliations:** 1 Department of Infectious Diseases, Hospital Universitari Vall d'Hebron, Barcelona, Spain; 2 Sexually Transmitted Infections Unit (Drassanes). Department of Infectious Diseases, Hospital Universitari Vall d'Hebron, Barcelona, Spain; 3 Microbiology Department, Hospital Universitari Vall d'Hebron, Barcelona, Spain

**Keywords:** amoebiasis, *Entamoeba histolytica* infection, outbreak, men who have sex with men, MSM, sexually transmitted disease

## Abstract

*Entamoeba histolytica* has been recently recognised as an emerging sexually transmissible pathogen in men who have sex with men (MSM), causing sporadic outbreaks in countries where it is not endemic. Here we report two closed clusters of invasive amoebiasis occurring in Barcelona, Spain, in October 2016 (four cases) and in January 2017 (four cases).

Four cases of intestinal amoebiasis in men who have sex with men (MSM) were diagnosed within two consecutive days in October 2016 and another four cases in a period of two weeks in January 2017 (three of them on the same day), in Barcelona (Spain). We provide a description of epidemiological and clinical features of these eight patients and discuss the characteristics of this potentially re-emerging infection in the MSM population. 

## Case Series

All eight patients were MSM and presented with proctocolitis to a referral clinic for sexually transmitted infections (STIs) in Barcelona (Table). Mean age at diagnosis was 41.5 years (range: 21–56 years). Four were HIV-positive, all with a CD4^+^- T cell count above 500/mm^3^. Regarding clinical manifestations, five patients had abdominal pain, four had diarrhoea with mucus, three had proctitis, two had dysentery, one had diarrhoea without mucus, and only one had fever. Mean duration of symptoms was 4 days (range: 2–10 days).

**Table:** Epidemiological information for two clusters sexually transmitted amoebiasis in men who have sex with men, Barcelona (Spain), October 2016 and January 2017 (n = 8)

Case	Age group (years), origin	Symptoms	Year of diagnosis	Microscopic analysis of stool	*Entamoeba histolytica* antigen in stool (ELISA)	Faecal cultures	*Entamoeba histolytica* serological test (ELISA)	HIV status (CD4, VL)	Concurrent STI	Response to treatment
A	50–59, UK	Diarrhoea with mucus, abdominal pain	2016	*Entamoeba* sp. trophozoites	Positive	*Shigella flexneri*	Negative	Positive (750, < 25)	No	Recurrence
B	30–39, UK	Fever, abdominal pain, dysentery, proctitis	2016	Negative	Positive	*Shigella flexneri*	Negative	Negative	Anorectal herpes simplex virus infection	Cure
C	20–29, The Netherlands	Dysentery, proctitis	2016	*Entamoeba* sp. trophozoites	Positive	Negative	Negative	Negative	Anorectal syphilis 1 month before	Cure
D	40–49, Spain	Proctitis	2016	Negative	Positive	Negative	Negative	Negative	No	Recurrence
E	20–29, Spain	Diarrhoea with mucus, abdominal pain	2017	Negative	Positive	Negative	NA	Negative	Rectal clahmydial infection	Cure
F	40–49, Uruguay	Diarrhoea, abdominal pain	2017	*Entamoeba* sp. trophozoites	Positive	Negative	NA	Positive (664, < 25)	No	Cure
G	30–39, Venezuela	Diarrhoea with mucus, abdominal pain	2017	*Entamoeba* sp. trophozoites	Positive	Negative	NA	Positive (520, 497.000)	Recent HIV infection	Cure
H	40–49, Spain	Diarrhoea with mucus, proctalgia	2017	*Entamoeba* sp. trophozoites	Positive	Negative	NA	Positive (1032, < 25)	Anorectal herpes simplex virus infection	Recurrence

CD4: CD4^+^ T cell count per mm^3^; M: male; NA: not available; STI: sexually transmitted infection; UK: United Kingdom; VL: viral load in RNA copies per mL.

Fresh stool mount microscopic examination was positive for *Entamoeba histolytica*/*dispar/moshkovskii* complex trophozoites in three patients. In all eight patients, *E. histolytica*-specific adhesion antigen was detected by ELISA (Entamoeba CELISA Path, Cellabs). Bacterial faecal cultures for *Salmonella*, *Shigella*, *Yersinia*, *Aeromonas*, *Campylobacter*, *Vibrio* and *E. coli* were done for all patients and were positive for *Shigella flexneri* in two. Serum detection of *E. histolytica* antibody IgG by ELISA (NovaLisa Entamoeba histolytica IgG, NovaTec Immunodiagnostica GmbH, Dietzenbach, Germany) was performed in four patients to exclude extraintestinal dissemination and was negative in all (absorbance index < 0.9). 

Surprisingly, no epidemiological relationship among any of the patients was found. The only common factor was that two of them had recently travelled to the United Kingdom (UK), and that all of them had practiced oral–anal sex. Seven patients had between 1 and 30 sexual contacts in the 3 months before diagnosis, and one reported 100 contacts. Contact tracing was only possible for four patients. One patient recalled two non-infected sexual contacts (microscopy and *E. histolytica*-antigen detection in the contacts were negative) and another sexual contact in the UK who had travelled to Brazil and been diagnosed with amoebiasis before him. Two patients had asymptomatic stable partners with a negative faecal screening, and one patient had three contacts that could be reached, two not infected and one with a positive faecal screening but asymptomatic. 

Five patients recovered after treatment with metronidazole followed by paromomycin, and three patients required repeated treatment due to poor adherence to paromomycin.

## Discussion

*E. histolytica* infection (EHI) can range from asymptomatic infection to invasive intestinal or extra-intestinal disease, with the most frequent manifestations being amoebic colitis, as in our eight cases, and liver abscess [1,2]. The diagnosis should be confirmed by the detection of *E. histolytica*-specific antigen in stool, to differentiate it from other non-pathogenic amoebas. Serological tests may help in the diagnosis of invasive amoebiasis [3], but its sensitivity can vary with the type and stage of disease [4] and they can be negative in acute infections, as it happened in the cases reported here.

Since the 1980s, EHI or amoebiasis has been increasingly reported among MSM [1,2,5], particularly those with HIV infection, in developed non-endemic countries such as Australia, Japan, Korea, and Taiwan [6-9]. Several studies that used serological tests, most of them from the 1980s, reported a seroprevalence of EHI ranging from 1% to 21% among MSM in several developed countries, a considerably higher seroprevalence than among heterosexual people (0–7%) [1,10-12].

Higher rates of EHI among homosexual men seem to be attributable to the practice of oral–anal sex [13,14], either directly or via sex toys or fellatio, and may reflect high-risk sexual behaviour and multiple exposures. This is supported by reports of high rates of intestinal sexually transmissible infections in MSM who visit sex venues [15]. Our cases had as risk factors the practice of oral–anal sex, patients A and E reported high risk sexual behaviours such as sex parties, and one patient had had sexual contact with a man from India. It is a limitation of this study that our patients were not systematically asked if they had practiced chemsex.

Hung et al. [6] found in Taiwan that invasive amoebiasis, high antibody titres in indirect haemagglutination and faecal colonisation with *E. histolytica* were more common in HIV-infected than in HIV-uninfected persons. In Japan, nearly 80% of the 600 cases of amoebiasis diagnosed every year occurred in MSM [16] and invasive amoebiasis was associated with syphilis and HIV infection [17-19]. Four of our patients had an HIV infection, one had also recently been diagnosed with syphilis and two had a co-infection with *S. flexneri,* another sexually transmissible enteric infection described in MSM [13,15]. It remains unclear whether HIV-related immunosuppression is a risk factor for amoebiasis or if this association may reflect a greater risk of sexual exposure. Our four patients with HIV had CD4^+^-T cell counts above 500/mm^3^ and only one had a detectable viral load, but five had other STIs. Thus amoebiasis was probably the result of high risk behaviours that are common in HIV-positive MSM. 

In Spain, endemic amoebiasis has been virtually eradicated in the last century after improvements in water infrastructure, and most cases are imported from endemic areas. To best of our knowledge, there are no previous published reports of cases of invasive amoebiasis in MSM in Barcelona. Given that a disease outbreak is defined by the World Health Organization as the occurrence of cases of disease in excess of what would normally be expected in a defined community, geographical area or season, we should consider these two clusters of EHI an outbreak in the MSM population [20]. It is noteworthy that two cases coinfected with *S. flexneri* had recently travelled to the UK where a prolonged outbreak of shigellosis among MSM had been noted [21]. Moreover, an increasing trend in shigellosis cases was also noted in MSM in Spain [22]. This may reflect the risk of enteric infections spreading across Europe through sexual transmission in such a highly mobile population, as it has happened with recent outbreaks of hepatitis A [23-25].

Outbreaks of amoebiasis like the one we report are of public health and clinical concern because EHI may be spread in the MSM population and can cause severe disease. Hence surveillance networks and notification systems are essential. In addition, asymptomatic infection should be tested for and treated because of its potential to progress to an invasive disease [4], although it can be difficult to carry out contact tracing in order to interrupt transmission. Moreover, there is a risk of further spread into non-MSM populations, as reported in Tokyo, where there were increased rates of *E. histolytica* seropositivity in heterosexual women [26], and in Canada, where a sexually transmitted cluster of amoebiasis in heterosexuals and female homosexuals were described [5]. If the need arises, molecular typing could be helpful to better define outbreaks. Periodic screening using molecular techniques in rectal swabs could be useful in preventing the spread of the infection, especially in MSM populations with high-risk behaviours. It has been shown to be a feasible and acceptable method for detecting gastrointestinal infections in previous studies [27].

## Conclusion

In order to prevent morbidity and transmission, physicians should be aware of amoebiasis as an emerging infection in the MSM population in non-endemic countries, with a greater risk in HIV-infected individuals. Therefore, MSM who are diagnosed with EHI or other enteric infections should be referred for STI and HIV testing, but unless the infection is recognised as being sexually transmitted, this may not happen. Moreover, information campaigns may target risk groups and relevant venues.
